# Institutional Needs

**Published:** 1913-08-09

**Authors:** 


### Institutional Needs.

### THE SURGICAL MANUFACTURING COMPANY'S CATALOGUE.

There was a time when a certain degree of sleepiness was a noticeable characteristic of leading London instrument-making firms. But the last ten years have changed all that, and assuredly no such reproach can be levelled against the Surgical Manufacturing Company, of 85 Mortimer Street, W., who have favoured us with a copy of their latest catalogue. The sections dealing with surgeons' instruments of every possible kind are sufficient proof of the fact that this firm is determined to keep the prominent position it has already won for itself; but to institutional managers Part 3, which deals with operating theatre furniture and fittings, ward lockers, sinks, bedsteads, and scores of similar hospital necessaries, will be of even greater interest. This business has suffered during the last two or three years from very severe foreign competition, and it is most gratifying to find that a London firm, all of whose goods are British made, cannot merely hold its own with our foreign rivals, but can go one better in pretty well every department.

